# Supplementary material for: Characterization of the ATPase FlaI of the motor complex of the Pyrococcus furiosus archaellum and its interactions between the ATP-binding protein FlaH
Source: PeerJ. 2018 Jun 18;6:e4984. doi: 10.7717/peerj.4984 (PMC6011876; doi:10.7717/peerj.4984)
Supplement: Supplemental Information 1 — Tables listing the primers, plasmids and strains used in this study. [file peerj-06-4984-s001.docx]

**SUPPLEMENTARY MATERIAL**

TABLE S1. **Plasmid used in this study**

| Plasmid | Relevant characteristics | Source |
| --- | --- | --- |
| pSVA 3116 | pETDuet-1 containing N-terminal His6 tagged *Pf*FlaI, Amp^R^. | (26) |
| pSVA 2167   \|  \|  \|  \| \| --- \| --- \| --- \| | pETDuet-1 containing N-terminal His6 tagged *Pf*FlaH. | (26) |
| pSVA 2176 | pETDuet-1 containing K39A mutation in *Pf*FlaH | (26) |
| pSVA 2177 | The K39A mutation *Pf*FlaH was created via round PCR on pSVA 2167 using primers 6001 and 6002. Amp^R^. | This study |
| pSVA 3140 | pETDuet-1 containing N-terminal StrepII-tagged *Pf*FlaI. The PCR product obtained using the 5118 and 5110 primers on pSVA 3116 was cloned using the NcoI and PstI sites. Amp^R^. | This study |
| pSVA 3146 | The E366A mutation in *Pf*FlaI was created via round PCR on pSVA 3140 using primers 5143 and 5144. Amp^R^. | This study |
| pSVA 3169 | pETDuet-1 containing N-terminal His6 tagged *Pf*FlaI-NTD. The PCR product obtained using the 5109 and 5185 primers on *P. furiosus* genomic DNA and was cloned using restriction sites EcoRI and PstI. Amp^R^. | This study |
| pSVA 3170 | pETDuet-1 containing N-terminal His6 tagged *Pf*FlaI-CTD. The PCR product obtained using the 5186 and 5110 primers on *P. furiosus* genomic DNA and was cloned using restriction sites EcoRI and PstI. Amp^R^. | This study |

TABLE S2. **Primers used in this study**. Relevant restriction sites are underlined.

| Primers | Sequence and characteristics | Source |
| --- | --- | --- |
| 5109 | 5’-GGGGAATTCGATGGCGGAAGTTATGTCAC-3’ | (26) |
| 5110 | 5’-GGGCTGCAGTCAGATTCTGAAGCTTAGTC-3’ | (26) |
| 5118 | 5’-GGGCCATGGGCTGGAGTCATCCACAATTTGAGAAGATGG  CGGAAGTTATGTCAC-3’ | This study |
| 5143 | 5’-CATTATCGTCGGTGCGATTAGAGGTGC-3’ | This study |
| 5144 | 5’- CCGCACCTCTAATCGCACCGACG-3’ | This study |
| 5185 | 5’-GCGGCTGCAGAGTTGCAGCGAACTTTCTTATTGTTGCAC-3’ | This study |
| 5186 | 5’-GAATTCGCCACTCAGCATAACTCAGTTAG-3’ | This study |
| 6001 | 5’-GCACGGATGAAAATGAATTAATGATTACAACATCGGGC-3’ | This study |
| 6002 | 5’-GCCCGATGTTGTAATCATTAATTCATTTTCATCCGTGC-3’ | This study |
